# Aquatic Interventions to Improve Motor and Social Functioning in Children with ASD: A Systematic Review

**DOI:** 10.1007/s40489-024-00464-z

**Published:** 2024-05-22

**Authors:** Patty van t Hooft, Janet Moeijes, Catharina Hartman, Jooske van Busschbach, Esther Hartman

**Affiliations:** 1 Center for Human Movement Sciences, University Medical Center Groningen, University of Groningen, Groningen, the Netherlands; 2 University Center of Psychiatry, University Medical Center Groningen, University of Groningen, Groningen, the Netherlands; 3 Lectorate of Human Movement, Health and Wellbeing, Departement Human Movement and Education, Windesheim University of Applied Sciences, Zwolle, the Netherlands; 4 Interdisciplinary Center Psychopathology and Emotion Regulation (ICPE), University Medical Center Groningen, University of Groningen, Groningen, the Netherlands

**Keywords:** Autism spectrum disorder, Aquatic activities, Swim activities, Motor skills, Social skills

## Abstract

Children with autism spectrum disorder (ASD) often have motor impairments. A promising strategy to improve motor and social functioning in children with ASD may be an aquatic intervention because of the properties of water. This systematic review investigated the characteristics and effects of aquatic interventions on motor and social skills in children with ASD. Searches in six databases on studies conducted between 2000 and 2023 resulted in 19 intervention studies involving 429 children aged 3 to 17 years with ASD. Best evidence syntheses and meta-analyses were used to evaluate the effects. Aquatic interventions guided by a combination of professionals in influencing behavior and in aquatic skills improved motor and social skills and significantly decreased autistic behavior in children with ASD.

## Introduction

Autism spectrum disorder (ASD) is a neurodevelopmental disorder, characterized by impairments in social interaction and communication and repetitive restrictive behaviors (American Psychiatric Association, 2013). ASD persists throughout the lifespan (Bölte et al., 2021; Lai et al., 2020). The prevalence of ASD across the world varies (Chiarotti & Venerosi, 2020); the World Health Organization (WHO) estimates the global prevalence of ASD to be one person in 160 (WHO, 2013).

Even though motor problems are not part of the formal classification criteria of ASD, about 80% of children with ASD are at risk of motor impairments (Bhat, 2021; Emck, 2014; Green et al., 2009; Hilton et al., 2012; Licari et al., 2020; Miller et al., 2021; Stins & Emck, 2018; Zampella et al., 2021). Motor skills provide the ability to produce efficient action to achieve an intended outcome by executing learned sequences of movement (Derikx et al., 2021; Staples et al., 2012). The less developed motor skills in children with ASD, in turn, hinder development in general (Adolph & Hoch, 2019; Bhat et al., 2022; Holloway et al., 2018; Pusponegoro et al., 2016). For example, motor impairments in children with ASD are associated with social developmental problems too (Bhat et al., 2022; Emck, 2014; Holloway et al., 2018; Ohara et al., 2019; Pusponegoro et al., 2016; Zampella et al., 2021). Reciprocal influences can explain these findings; that is, motor skills are often practiced in the context of social interaction, and social interaction through which social skills are learned is easier if a child has adequate motor skills (Adolph & Hoch, 2019; Casartelli et al., 2016; Derikx et al., 2021; Ohara et al., 2019).

Improving the motor skills of children with ASD may help children perform better in motor activities which may, in turn, decrease the limitations in social interactions (Bhat, 2021; Ketcheson et al., 2021; Licari et al., 2020; Wang et al., 2022; Zampella et al., 2021). One strategy to improve motor and social functioning in children with ASD may be an aquatic intervention (Aleksandrovic et al., 2016; Mortimer et al., 2014; Murphy & Hennebach, 2020; Ruggeri et al., 2020).

According to the dynamic systems theory, motor skill development is a result of a self-organizational process, influenced by the interaction between the environment, task, and individual components. To improve motor skills, any of the three can be manipulated. If one component changes, the other two are adapting to that change. While adapting to the change, a child with ASD may develop new movement patterns (Colombo-Dougovito, 2017; Lee & Porretta, 2013). Since children with ASD have problems in social and communicational aspects, it seems difficult to effectively instruct children with ASD to do tasks frequently enough to improve motor development. Therefore, an aquatic intervention could be an effective strategy to improve motor and social functioning in children with ASD because the aquatic context provides implicit task manipulation for motor development because of the physical principles of water (Becker, 2009; Cha et al., 2017; Gamper & Lambeck, 2011; Muñoz-Blanco et al., 2020). The specific properties of water, like buoyant force, water resistance, and turbulence through waves and currents, demand constant attention from the motor system. Being in the water provides a “natural” balancing task (Bommer & Lambeck, 2011; Lambeck & Gamper, 2011). This is why most aquatic interventions in children start with a water adjustment phase, where movement is mostly vertical so previous land-related motor skills can be used. In this adjustment phase, the child learns to respond independently, automatically, and appropriately to all the situations in the water (Lambeck & Gamper, 2011). These adjustment skills, described often as mental adjustment, are seen as conditional for learning aquatic skills, where horizontal movement is introduced (Barrett & Maes, 2021; Lambeck, 2015; Lambeck & Gamper, 2011).

Another positive aspect of aquatic interventions is that water ensures that impaired balance and coordination do not lead to falling risks and failure experiences (Lambeck, 2015; Vasile & Stănescu, 2013). The resistance of the water slows down a fall. Balance and relief reactions are thus given time to eliminate the balance disturbance (Becker, 2020; Bommer & Lambeck, 2011; Fatorehchy, 2019; Veldema & Jansen, 2021), so children with ASD will be able to experience success rather than failure. Even if there is a fall, the effects are less or no burden on the musculoskeletal system and will not cause pain. Water also offers a range of sensory experiences that implicitly appeal to the stimulation of information processing (Herold et al., 2016). Children with ASD might benefit from more movement experience to improve their motor skills in a safe aquatic environment. Therefore, moving in water seems to offer good opportunities to practice, experience, discover, and develop motor skills (Cole & Becker, 2004; Lambeck, 2015; Lambeck & Gamper, 2011; Vonder Hulls et al., 2006).

Aquatic interventions are found in different contexts, such as therapy or swimming instruction. In general, therapy treats symptoms that cause problems, while education or instruction is intended for learning, the latter usually referred to as learning to swim. Aquatic therapy can aim at land- and water-related outcomes, while interventions on swimming instruction most likely have goals on water-related outcomes. Therefore the intended goals, outcomes, and the ways of intervening in aquatic interventions can differ. This could explain why previous reviews indicated that there is accumulative evidence about aquatic interventions related to increased motor and social skills in children with ASD, but that the outcomes are heterogeneous (Hynes & Block, 2023; Mortimer et al., 2014; Ruggeri et al., 2020). Before synthesizing the outcomes of the studies on aquatic interventions for children with ASD, it is necessary to get a thorough understanding of these interventions by evaluating not only the outcomes but also the types of aquatic interventions, their goals, and how these goals were measured. Understanding which components of aquatic interventions ensure optimal outcomes will enable practitioners to enhance their aquatic interventions.

Therefore, this review aims to investigate effects of aquatic interventions in children with ASD, by focusing on intervention outcomes related to motor skills (water-related or land-related) and social skills as well as autistic behavior, and on the description of the characteristics of the interventions that might be related to their effectiveness, characteristics consisting of instructor-child ratio, exposure duration, interventionist, aims, and learning support. Our approach is to (1) identify appropriate studies, (2) characterize the interventions and their substantiation, (3) identify the outcomes, and (4) combine—if possible—outcomes for a best evidence analysis by pooling the data to increase the sample size. To the best of the authors’ knowledge, no previous reviews are performing best evidence syntheses on motor and social outcomes in aquatic interventions for children with ASD.

## Method

This systematic review was conducted using the guidelines from the Preferred Reporting Items for Systematic Review and Meta-analysis (PRISMA) (Moher et al., 2009). Before conducting the study, we established study procedures including search strategy, inclusion criteria, and data extraction specifications.

### Search Strategy/Information Sources

To conduct this review, six databases were searched: CINAHL, Clinicaltrials.gov, Cochrane, Eric, PEDro, and PubMed. The MESH terms used were Autism Spectrum Disorders, Aquatic, Swim, or Hydrotherapy. Filters were applied for language (English, German, Dutch). The first search period was between November 19 and 23, 2021, and the last searches were done in March 2023.

### Eligibility Criteria

Randomized controlled trials (RCT), controlled clinical trials (CCT), crossover and cohort studies on aquatic or swim interventions enhancing motor skills or social skills, or a combination of both, in children aged between 2 and 14 years diagnosed with ASD, with pre-and post-data on at least one outcome, were included in this review. This broad search strategy was chosen to get a thorough overview and insight on the (potential) outcomes of a wide variety of aquatic interventions. Conference papers, abstracts, and dissertations were excluded to ensure that only high-quality peer-reviewed research was included.

### Data Selection Process

After removing duplicates, two researchers (first mentioned authors) worked independently in Rayyan, a Web App for systematic reviews (Ouzzani et al., 2016), to determine which studies met the eligibility criteria. First eligibility was screened on the titles and second on the abstracts. After each of these screening phases, disagreement about whether a study should be included was solved by discussion until a consensus was reached. The remaining studies were assessed for eligibility based on the content of the full article by the first author. Appendix Figure 3 shows the PRISMA flow diagram of the selection process.

### Data Items

The outcomes for which data were collected were as follows:

1. Study design, for weighing the results and risk of bias
2. Participant characteristics (number, sex, and age)
3. Intervention characteristics such as instructor-child ratio, exposure duration, interventionist, aims and learning support
4. Data on the type of outcomes (motor/social skills) and the measurement instruments used to check the homogeneity of the methods and measurements
5. The statistical outcomes of the between-group and within-group results to be able to compare and analyze these results

Tables 1 and 2 present these data items.

**Table 1:** General characteristics of the included studies

Authors	Study design	PEDro	Participants *N *= (boys/girls) Age (SD)	Intervention/type of pool	Ratio instructor-child	Exposure	Outcome
Alaniz et al. (2017)	Single group pre-post test	Poor	*N*=7 (6/1) Age: 3–7 year M: 6.095 (0.236) Mild-severe ASD	Occupational therapy based aquatic therapy/ Indoor heated therapy pool	1:2	8 weeks (*n*=7), 16 w (*n*=6) 24 w (*n*=5) 1 h a week	Water safety skills Social skills
Ansari et al. (2021)	RCT	Fair	*N*=30 (30/0) Age: 8–14 *N*=10 Kata A:10.80 SD 2.14 *N*=10 aquatic intervention A: 10.60 SD 2.50 *N*=10 control A: 10.80 SD 2.14	1 group Halliwick-based aquatic intervention/indoor public pool 1 group Kata Control group: therapy as usual	1:2	10 weeks 2 sessions per week 60 min	Balance
Caputo et al. (2018)	CCT	Good	*N*= 26 (17/9) Experiment *n*=13 (11/2) Age:8.3 SD 2.3 Control *n*=13 (6/7) A:7.7 SD 2 No comorbidity	CI-MAT Multisystem Aquatic Therapy/Community pool Control: therapy as usual	1:1 Phases 1 & 2 1:4–6 Phase 3	Total 10 months, 96 session The first 2 phases 45 min once a week 3rd phase twice a week 45 min	Autistic symptoms Adaptive behavior Aquatic skills (exp group only)
Chu and Pan (2012)	RCT	Fair	*N*=42 *N*=21 ASD (20/1) *N*=21 TD (6/15) Age ASD 6–11 Age TD 6–11	After-school aquatic program three instructional conditions regarding support provided to children with ASD across teacher-directed, peer and sibling-assisted, and voluntary peer support conditions (control)/local indoor hydrotherapy pool	1:1 pair In a group of two pairs	16 weeks, twice a week 60 min	Interaction behaviors and aquatic skills
Ennis (2011)	Single group pre-post	Poor	*N*= 6 (not described) Age: 3–9 5 dropouts not included	Physical therapy-based aquatic program/ therapy pool in local facility	1–2:1 In groups 4–6	10 weeks once a week 60 min 3 groups in 2 semesters	Aquatic skills Quality of life
Fragala-Pinkham et al. (2011)	CCT	Fair	*N*=12 (11/1) Age: 6–12 Experiment *n*=7 (6/1) A: 9.6 (2.6) Control *n*=5 (5/0) Age:9.6 (1.3)	Group aquatic exercise program Control: therapy as usual/YMCA community pool	1:2	14 weeks twice per week for 40 minutes	Swimming skills (both control & exp. group) WSC(exp group only) Cardiorespiratory endurance Muscular endurance mobility skills satisfaction
Güeita-Rodríguez et al. (2021)	Mixed methods Single group pre-post test	Poor	*N*=6 (5/1) 3 dropouts not included Age: 7.17 (±1.60)	Aquatic physical therapy using learning strategies/institutional pool	1:1	60 min twice a week over seven months	Perceived competence Adaptation to water Quality of life Behavior patterns Social communication Emotions & acceptance-rejections
Lawson et al. (2014)	Pre-post design	Poor	*N*= 42 (39/3) Age: 4–15	Sensory-enhanced swim training/university or community pool	1:1 or 2:3	8 sessions of 30 min Summer twice a week Fall once a week	Swim skills Enjoyment Participating
Marzouki et al. (2022)	RCT	Fair	*N*=22(20/2) Age: 6.3 (0.5) TAT *n*=8 (7/1) GAT *n*=8 (8/0) Control *n*=6 (5/1), 2 dropouts not included	Technical aquatic activities Game-based aquatic activities / local indoor swimming pool Control TAU	1:2	8 weeks 2× 50 min	Gross motor skills Stereotyped behavior Emotion regulation
Mills et al. (2020)	Crossover clinical trial	Fair	*N*= 8 (6/2) Age: 6–12 years M: 8.72 (1.99)	Play-based physiotherapeutic hydrotherapy/both heated hydrotherapy or outdoor pool Participants were their own control. 4 weeks of no intervention before or after the intervention	1:1	Once a week for 4 weeks 45 min	Behaviors are related to mental health and well-being Social, behavioral, and emotional outcome Enjoyment
Munn et al. (2021)	Efficacy study Single-group pre- and post-test	Poor	*N*=86 (75/11) Age: 3–16 M: 8.84 Comorbidity ADHD *n*=17 (16/1)	Group “I can swim program”/different unspecified pools	1:1 Group extra 2:6	5 days, daily 45min (A:3-7) 60 min (A:8+)	Aquatic skills
Oriel et al. (2020)	Pre-post	Poor	*N*=13(6/7) 7.7 (-)	Aquatic social competence program/heated indoor college pool	1:1	8 weeks 1x60 min.	Social competence
Pan (2010)	Cross-over clinical trial	Fair	*N*= 16 (16/0) Age:5–10 exp *N*=8 A:7.27 ± 1.25 control *N*=8 A: 7.20 ± 0.89	Water exercise swimming program /local indoor hydrotherapy and swimming pool Control: Therapy as usual, after 10 weeks change from control to experiment and vice versa	1:2	10 weeks Twice a week 90 min	Aquatic skills Social behavior
Pan (2011)	Cross-over clinical trial	Good	ASD *N*=15 (15/0) group 1: *n*=7 Age:9.31 (1.67) group 2 *n*= 8 Age: 8.75 (1.76) Sibling no-ASD *N*=15 gr 1 *n*=7 (1/6) Age: 8.89 (1.98) gr2 *n*=8 (4/4) Age: 7.39 (2.94)	Aquatic program utilizing the principles of motor learning and physical fitness learning/local indoor hydrotherapy pool Control: therapy as usual	1:2	14 weeks twice a week 60 min	Cardiovascular endurance Muscular strength/endurance Range of motion Body composition Aquatic skills
Yanardag et al. (2013)	Single group pre-post test	Poor	*N*=3 ( 2/1) Age: 6–8	Video prompting aquatic play/university indoor pool	1:1	12 weeks 3 times a week 1 h	Motor skills Video prompting effect
Yilmaz et al. (2004)	Pre-post single-case design	Poor	N=1 (1/0) Age: 9	Halliwick method/type of pool not mentioned	1:1	10 weeks 3 times a week 1 h	Physical fitness Motor skills Orientation in water Autistic behavior
Yılmaz et al. (2005)	Multiple observational designs	Poor	*N*=4 (4/0) Age:7–9	Play specific activities in water (snake, Kangaroo, cycle)/ university indoor pool	1:1 in a group together (4:4)	10 weeks 3 times a week 1hour	Learning 3 specific aquatic skills
Yilmaz et al. (2010)	A multiple baseline design across subjects	Poor	*N*=3 (3/0) Age:9	Teaching simple progression swimming skills/ university indoor pool	1:1	10 weeks 3 times a week 1hour	Learning Halliwick-based simple progression skills
Zanobini and Solari (2019)	CCT	Fair	*N*=25 (19/6) Age:3–8 exp *N*=13(10/3) A: 5.6 sd 1.3 Control *N*=12 (9/3) A:5.4 sd 1.5	Group aquatic program “Water as a mediator of communication”/community and rehabilitation center pool Control: different forms of psychoeducational or sports laboratories.	1:1 in groups of 6 or 7	Once in 2 weeks 12 sessions of 30 min	Aquatic Skills: (exp group only) interpersonal skills Autistic mannerisms
*A* age, *M* mean, *SD* standard deviation, *TD* typical development/neurotypical

**Table 2:** Best evidence syntheses

Outcomes	Effectiveness and safety* Based on RCTs and CCTs	Author (PEDro score)	N** of participants (studies)	Best evidence synthesis	Supported by within-group/descriptive effects
*Motor aspects*					
Adjustment to the aquatic environment	Evidence suggests a medium to large effect size with a significant difference in stage 2 of the HAAR (Pan, 2011). Pan (2010) found no significant differences	Significant: Pan (2011) (6) Pan, 2010 (4) NS Chu and Pan (2012) (5)	52 (3)	Moderate evidence	Within-group results: 4 studies found significant changes in the intervention group (Caputo et al., 2018; Chu & Pan, 2012; Pan, 2010; Zanobini & Solari, 2019) and one pre-post study had non-significant change but mentioned large effect size (Güeita-Rodríguez et al., 2021) Not statistical analyzed descriptive positive results reported by Yılmaz et al. (2005), Ennis (2011) and Yanardag et al. (2013)
Aquatic skills	Only one study found significant differences between groups (Pan, 2011). No significance in (Chu & Pan, 2012), Fragala-Pinkham et al., (2011), (Pan, 2010). Moderate to large ES were found	Significant Pan (2011) (6) Pan (2010) (4) NS Chu and Pan (2012) (5) Fragala-Pinkham (4)	64 (4)	Moderate evidence	Within-group results: Positive significant changes by Caputo et al. (2018), Chu and Pan (2012), Fragala-Pinkham et al. (2011), Pan (2010); Zanobini and Solari (2019) and Alaniz et al. (2017) Descriptive positive results by Ennis (2011), Lawson et al. (2014); Lawson et al. (2017), Munn et al. (2021), and Yilmaz et al. (2010)
Land-related motor skills	Ansari et al. (2021) found a significant difference with a large effect size in both static and dynamic balance	Significant Ansari (4) Marzouki (5)	42 (2)	Moderate evidence	Descriptive, not statistical analyzed positive results by Yanardag et al. (2013)
Physical Fitness	Pan (2011) found significant differences and Fragala-Pinkham et al. (2011) found none. The effect size in the latter study is medium to large suggesting an increase in physical fitness in the intervention group. But small effect sizes on cardiovascular and mobility	Significant Pan (2011) (6) NS Fragala-Pinkham (4)	27 (2)	Limited evidence	Descriptive, not statistical analyzed positive results by Yilmaz et al. (2004)
*Social aspects*					
Social functioning	Chu and Pan (2012) found positive significant differences in social interaction and activity engagement. Pan (2010) found no significant difference in social competence, only on the academic behavior scale (1 of 3 items), a significant difference was found in favor of the experiment group. Caputo et al. (2018) found significant differences on the daily living scale (1 of 4 items of the VABS). Zanobini and Solari (2019) found significant differences in social relating, (1 of 5 items of the ABC) and social motivation (1 of 5 scales SRS)	Significant Chu and Pan (5) Partly significant Caputo (5) Zanobini (4) Pan (2010) (4)	88 (4)	Limited evidence	Within **intervention** group results: positive significant changes by Caputo et al. (2018) on all subscales VABS, Zanobini and Solari (2019) on total SRS and social motivation Pan (2010) on the total scale of social competence, Oriel et al. (2020) on social competence total in Home and Community Behavior Scale (HCSBS) Within the **control** group, Zanobini and Solari (2019) found a positive significant difference in the total score of SRS and Caputo et al. (2018) 2,020,222 on the total VABS scale but not the on daily living and social abilities scales. Alaniz et al.(201,721,072,017) did not find significant changes. Descriptive, not analyzed, positive results or large effect size by Ennis (2011); Güeita-Rodríguez et al. (2021)
Autistic behavior	Pan (2010) found significantly decreased anti-social behavior. Caputo et al. (2018) found a significant difference in emotional response, adaptation to change, and activity level (3 of 15 subscales) Zanobini and Solari (2019) found a large effect size on the ABC and a significant decrease in autistic mannerisms on the SRS	Significant Pan (2010) (4) Marzouki (2022) (5) Partly significant Caputo (5) Zanobini (5)	89 (4)	Limited evidence	Within experiment group results: Caputo et al. (2018) found significant changes in 13 of the 15 subscales (CARS) where the control group had none. Zanobini and Solari (2019) found significant changes in total ABC scores for the experimental and control groups although the control group only in the body and object use scale, while the experimental group showed significant changes on all scales except language. Mills et al. (2020) found significant changes in the score of the total problems scale of the CBCL but not specific in the externalizing problems domain Not statistically analyzed, positive results by Yilmaz et al. (2004) on stereotypical behavior
*None of the included RCTs and CCTs reported adverse effects; **N = participants with ASD. *NS* not significant					

### Level of Evidence

The Physical Evidence Database Scale (PEDro) was used to evaluate the methodological quality of the included studies (Moseley et al., 2020). This scale measures the internal validity of the studies, including the level of bias. The total score ranges from 0 to 10 points. Scores of 0–3 are considered “poor,” 4–5 “fair,” 6–8 “good,” and 9–10 “excellent” (Cashin & McAuley, 2019). The first and second authors independently scored the articles with the PEDro Scale. Differences in outcomes were discussed and third and fifth authors were consulted to reach consensus. Appendix Table 3 contains the results of the quality assessment with the PEDro scale.

### Effect Measures

Due to the heterogeneity of the motor outcomes, these were divided into motor skills measured in the water, water-related motor skills, and motor skills measured outside of the pool, land-related motor skills. The water-related motor skills were subdivided into water adjustment and aquatic skills because the motor skills required differ from each other. The social outcomes were divided into general social skills and social aspects of autistic behavior.

A random-effect meta-analysis was conducted using RevMan 5 for social outcomes. The first two authors independently screened whether studies were eligible for a meta-analysis on outcomes related to social skills or autistic behavior and, after consultation, selected relevant (sub)scales and their statistics for the meta-analyses. Because of the incompleteness of data, the standard deviation (SD) of the post-measurement outcome was used as the standard deviation of the difference (Higgins et al., 2019).

To assess certainty, the best evidence syntheses on the PEDro scores were used for the synthesis of evidence of all the included studies with back-up from within-group results. The results of the studies with control groups were stratified into four groups indicating the level of evidence. Level 1 means strong evidence, substantiated in multiple relevant high-quality RCTs with a PEDro-score of at least 4 points. Level 2 means moderate evidence which is substantiated by one high-quality RCT and more relevant low-quality RCTs (≤ 3 points) or one controlled clinical trial (CCT) of high quality. Level 3 means limited consistent evidence in one or more relevant low quality RCTs. Level 4 means no evidence was found or the results were conflicting (van der Velde et al., 2007; Van Peppen et al., 2004; Van Tulder et al., 1999).

## Results

### Study Selection

After the screening process, 19 articles, all written in English, were eligible for this review. All studies except two (Mills et al., 2020; Oriel et al., 2020) reported on motor outcomes. Nine studies reported an outcome on both motor and social skills (Alaniz et al., 2017; Caputo et al., 2018; Chu & Pan, 2012; Ennis, 2011; Güeita-Rodríguez et al., 2021; Marzouki et al., 2022; Pan, 2010; Yilmaz et al., 2004; Zanobini & Solari, 2019). Outcomes on motor skills only were found in nine studies (Ansari et al., 2021; Fragala-Pinkham et al., 2011; Lawson et al., 2014; Munn et al., 2021; Pan, 2011; Yanardag et al., 2013; Yılmaz et al., 2005; Yilmaz et al., 2010).

Eight studies used a control group, three for comparing motor skills only, two studies for comparing social skills only, and three studies on both motor and social skills. The quality of evidence of these studies varied from good (1) to fair (7); see Appendix Table 3. None of these studies had blinded subjects, therapists, or assessors. The other 11 studies scored fair to poor, due to lack of controls, and those were not included in the best evidence syntheses. Table 1 shows the general characteristics of each included study.

Appendix Table 4 shows that seven of the studies were funded by a combination of public and non-profit organizations, four explicitly stated that they had no external funding, and eight studies did not disclose their funding.

### Participants

The total number of participants in this review was 465 (72 girls) of which 429 (47 girls) were children with ASD. In two studies (Chu & Pan, 2012; Pan, 2011), children without ASD participated besides children with ASD. The average percentage of boys was 90% overall within the ASD participants, with one study that did not report the sexes of the participants (Ennis, 2011). The age in all the studies ranged from 3 to 17 years old. The number of participants varied from one (Yilmaz et al., 2004) to 86 children (Munn et al., 2021). Only one study (Marzouki et al., 2022) used a power analyses to estimate the necessary sample size.

Not in all studies the level of severity of the ASD symptoms was described. Three studies included high-functioning children with ASD (Fragala-Pinkham et al., 2011; Pan, 2010, 2011). The other 15 studies mentioned diverse ASD severity levels varying from mild to moderate.

Most studies (*n* = 15) did not require previous aquatic interventions for eligibility and reported participants with mixed baseline aquatic skills. One study required 3 months of aquatic therapy experience for eligibility (Güeita-Rodríguez et al., 2021). In one study, all participants had experienced previous water interventions but it is unclear if that was a requirement for inclusion (Ennis, 2011).

In almost all studies, participants with physical limitations were excluded except for Ennis (2011) who included two participants with neuro-motor disorders, and Mills et al. (2020) with one participant having an additional medical diagnosis affecting physical capabilities.

In five of the eight controlled studies, there was one intervention and one control group where the participants in the control group received treatment as usual (TAU). The control group of Zanobini and Solari (2019) received different forms of sports and therapies. Ansari et al. (2021) compared three groups, one control group that received TAU, one group that received a Kata intervention, and one group received an aquatic intervention. Chu and Pan (2012) compared three groups who were all offered the same aquatic intervention but with different levels of social support from peers or siblings. Marzouki et al. (2022) also compared three groups, one that received technical aquatic training (TAT), one group game-based aquatic training (GAT), and the control group TAU.

### Interventions

All studies based their intervention on the fact that ASD is associated with delayed or reduced quality of motor development, and the assumption that improved motor skills can contribute to improving other physical, social, emotional, and cognitive developmental domains. Improving motor skills in general was the aim in the studies of Ansari et al. (2021), Caputo et al. (2018), Ennis (2011), Marzouki et al. (2022), Pan (2011), Pan (2010), and Yilmaz et al. (2004), whereas Ennis (2011) and Chu and Pan (2012) aimed at functional skills. Improving motor skills related to increased physical activity was the aim of Fragala-Pinkham et al. (2011), Lawson et al. (2014), and Yanardag et al. (2013). Improving motor skills related to water safety was found in the studies of Alaniz et al. (2017) and Munn et al. (2021). Aquatic interventions aimed at reducing autistic mannerisms were researched by Caputo et al. (2018), Marzouki et al. (2022), Pan (2010), Yılmaz et al. (2005), Yilmaz et al. (2010), and Zanobini and Solari (2019). Increasing the quality of life was the aim of the intervention of Ennis (2011), Mills et al. (2020), and Güeita-Rodríguez et al. (2021), the latter also aimed at social competence as did Oriel et al. (2020). Social interaction was the aim of the intervention by Alaniz et al. (2017) and Chu and Pan (2012) and social behavior by the intervention of Caputo et al. (2018), Pan (2010), and Zanobini and Solari (2019).

Most studies (*n* = 12) used “aquatic” to describe the intervention, four used “swim,” and the other three used “hydrotherapy,” “water,” or “Halliwick.” Additions to the intervention descriptions were “therapy-based,” of which one study used occupational therapy (OT), three studies physical therapy (PT), and one aquatic therapy (AT). In three studies, the interventions were characterized as “game” or “play-based,” in two studies as “exercise-based interventions” and one as social competence program.

The interventions were offered or supervised by physiotherapists (four studies), certified or qualified swim trainers (four studies), instructors (four studies) which in three studies were described as trained, experts/instructors specifically trained in the offered intervention, occupational therapists (two studies), one in combination with a language pathologist the other with a recreational therapist, researchers with degrees in special needs and aquatic experience (two studies), water confident educators specialized in special needs (one study), and students (four studies). The instructor/therapist-child ratio was mostly one on one (13 studies) or one on two (6 studies), even in group-based interventions.

The intervention exposure varied between 1 week (Munn et al., 2021) and 10 months (Caputo et al., 2018). Exposure time per session varied from 30 min (three studies) to 90 min (one study). The most common was a session duration of 60 min (12 studies). The shortest overall duration was 3 h in 4 weeks (Mills et al., 2020) and the longest was 72 h in 10 months (Caputo et al., 2018). The frequency of the intervention varied from daily (Munn et al., 2021) to once in 2 weeks (Zanobini & Solari, 2019). Most studies had a frequency of twice a week, whereas two studies varied their frequency in different phases (Alaniz et al., 2017; Caputo et al., 2018) or different seasons (Lawson et al., 2014).

Regarding learning and behavioral support, all studies except Lawson et al. (2014) described their interventions as structured or predictable. In most studies, the structure is shaped by a fixed sequence in the sessions. In addition to structured sessions, the following supports have been mentioned: physical support in all studies, sensory support (Alaniz et al., 2017; Güeita-Rodríguez et al., 2021; Lawson et al., 2014), visual support (Munn et al., 2021; Oriel et al., 2020), parental support (Ansari et al., 2021; Caputo et al., 2018; Ennis, 2011), prompting (Güeita-Rodríguez et al., 2021; Yanardag et al., 2013; Yılmaz et al., 2005; Yilmaz et al., 2010), support via Treatment and Education of Autistic and related Communication Handicapped Children (TEACCH) method (Chu & Pan, 2012), peer-assisted support (Chu & Pan, 2012; Pan, 2011; Zanobini & Solari, 2019), support via principles of motor learning (Güeita-Rodríguez et al., 2021; Pan, 2011), encouragement (Alaniz et al., 2017; Munn et al., 2021), and attachment theory (Caputo et al., 2018).

The methodologies used in the interventions vary from Halliwick concept (Ansari et al., 2021; Chu & Pan, 2012; Güeita-Rodríguez et al., 2021; Marzouki et al., 2022; Pan, 2010, 2011; Yanardag et al., 2013; Yılmaz et al., 2005; Yilmaz et al., 2004, 2010) to adapted existing swim skill program methodology (Fragala-Pinkham et al., 2011; Lawson et al., 2014; Munn et al., 2021) to intervention-specific methodology (Alaniz et al., 2017; Caputo et al., 2018; Ennis, 2011; Mills et al., 2020; Oriel et al., 2020; Zanobini & Solari, 2019).

The interventions took place in different pool facilities (see Table 1). Four studies (Lawson et al., 2014; Mills et al., 2020; Munn et al., 2021; Zanobini & Solari, 2019) used more than one type of pool for conducting their interventions. The pool facilities varied from therapy pools (five studies), community or local swimming pools (five studies), university swimming pools (five studies), institutional pools (one study), and heated outdoor pools (one study). One study did not provide information on the pool facility. Only the studies of Alaniz et al. (2017), Mills et al. (2020), and Oriel et al. (2020) mentioned the water temperature.

### Outcomes on Motor Skills

Outcomes on motor skills were measured in 17 studies, with 13 different measurement instruments, 7 instruments on water-related and 6 on land-related motor skills. All outcomes on motor skills were reported in terms of water-related motor skills (15 studies *n* = 270), except Ansari et al. (2021) and Marzouki et al. (2022) who reported only land-related outcomes (*n* = 55). Yanardag et al. (2013) reported on both land- and water-related skills and three studies (*n* = 28) had an extra outcome on physical fitness (Fragala-Pinkham et al., 2011; Pan, 2011; Yilmaz et al., 2004). Table 2 displays best evidence synthesis for these outcomes.

For *adjustment to the aquatic environment*, a significant between-group difference favoring the intervention group was found by Pan (2011) and Pan (2010). In the study of Chu and Pan (2012), no such difference was found, but significant within-group changes were reported. These outcomes lead to level 2, moderate evidence for water adjustment. This evidence is substantiated by significant changes within the intervention group reported in two studies without a control group on water-related outcomes (Caputo et al., 2018; Zanobini & Solari, 2019). One study, lacking a control group, reported no significant changes within the group (*n* = 3) but mentioned a large effect size (*r* = 0.531). Three studies, also without a control group, did not provide statistical analyses (Ennis, 2011; Yanardag et al., 2013; Yılmaz et al., 2005) but their results were interpreted by the authors as an improvement of water adjustment.

Between-group differences in *aquatic skills* were reported in four studies. Pan (2010) and Pan (2011) found positive significant differences in favor of the intervention in respectively all phases and phases IV and V of the Humphries’ Assessment of Aquatic Readiness (HAAR). Chu and Pan (2012) and Fragala-Pinkham et al. (2011) did not find any significant differences between intervention and control group although all reported significant positive changes within the intervention groups (Chu & Pan, 2012; Pan, 2010) or a large effect size (*r* = 0.66) (Fragala-Pinkham et al., 2011). For clarity, in the study of Chu and Pan (2012), all three groups were offered an aquatic intervention but with varying forms of support. Together, these outcomes lead to level 2, moderate evidence for increasing aquatic skills. This positive evidence is substantiated by within-group positive significant changes in *aquatic skills* in the intervention group reported in three studies without a control group (Alaniz et al., 2017; Caputo et al., 2018; Zanobini & Solari, 2019). Six other studies did report progression in the aquatic skills of the intervention group, but did not present statistical analyses (Ennis, 2011; Lawson et al., 2014; Munn et al., 2021; Yılmaz et al., 2005; Yilmaz et al., 2004, 2010).

On land-related motor skills, two studies reported significant differences. Ansari et al. (2021) found significant differences on *static balance* and on *dynamic balance skills* in favor of the groups that received the aquatic or the Kata intervention compared to the group that received TAU. Both aquatic interventions groups, technique and game based, of Marzouki et al. (2022), showed significant improvement in locomotor and object control skills compared to the control group. Together, these outcomes lead to level 2, moderate evidence. Positive evidence on progression in manual dexterity, aiming and catching, and dynamic and static balance, was also reported in the single-group pre-post study of Yanardag et al. (2013).

On *physical fitness*, Pan (2011) found significant difference between the groups regarding muscular endurance and the 30-s curl-ups. Fragala-Pinkham et al. (2011) found no significant differences between both groups in physical fitness outcomes but reported a large effect size (*r* = 0.90–1.28) within the intervention group. One single case study (Yilmaz et al., 2004) reported increased physical fitness. All together resulted in level 3, limited evidence for this outcome.

### Outcomes on Social Skills

As shown in Table 1, 11 studies covering 151 children with ASD reported outcomes in social skills (Alaniz et al., 2017; Caputo et al., 2018; Chu & Pan, 2012; Ennis, 2011; Güeita-Rodríguez et al., 2021; Marzouki et al., 2022; Mills et al., 2020; Oriel et al., 2020; Pan, 2010; Yilmaz et al., 2004; Zanobini & Solari, 2019) using 13 different measurement instruments. Significant between group differences on *social interaction* were found in the study of Chu and Pan (2012). Three studies found significant *increased social behavior* on subscales of the measurement instrument in favor of the experiment group (Caputo et al., 2018; Pan, 2010; Zanobini & Solari, 2019). Four pre-post studies (Ennis, 2011; Güeita-Rodríguez et al., 2021; Mills et al., 2020; Oriel et al., 2020) described positive change within the group by medium to large effect sizes in social skills. Alaniz et al. (2017) reported no significant change in social skills within the intervention group. These outcomes result in level 3, limited evidence.

Pan (2010) and Marzouki et al. (2022) found respectively significant decreased *anti-social behavior* and *stereotype behavior* in favor of the intervention group. Two other controlled studies (Caputo et al., 2018; Zanobini & Solari, 2019) found significant decreased *autistic behavior* on some subscales of their measurement instrument and both studies reported significant positive change within the experimental group on most subscales. These findings result in level 3, moderate evidence.

In Table 2, both between and within-groups significant differences in social skills and autistic behavior are displayed for all included studies.

### Meta-analyses on Social Functioning and Autistic Behavior

With three controlled studies (*n* = 67) reporting on social skills and four on autistic behavior (*n* = 89) and a fairly homogeneous intervention, it was possible to conduct two meta-analyses focusing on these outcomes. For the meta-analysis on social skills, data from the Social Abilities Scale from the Vineland Adaptive Behavior Scales (VABS) (Caputo et al., 2018), the total social competence scale from the School Social Behavior Scale-2 (SSBS-2) (Pan, 2010), and the data from the total Social Responsiveness Scale (SRS) (Zanobini & Solari, 2019) were used.

**Fig. 1:**
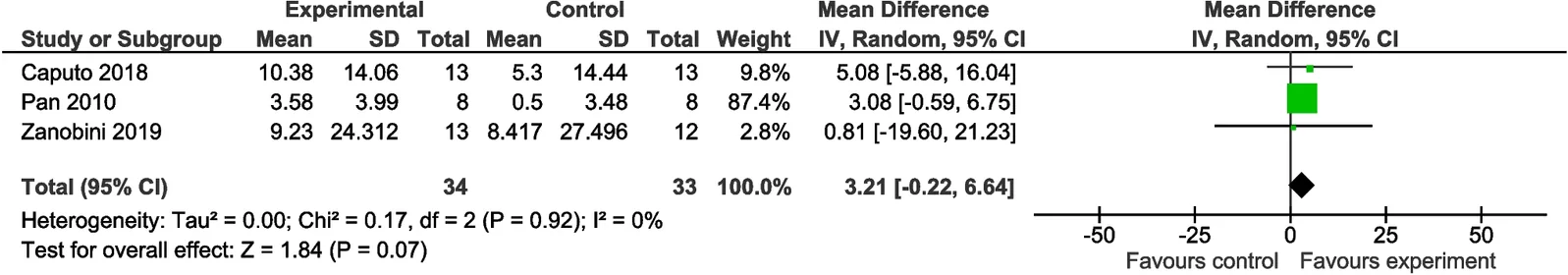
Meta-analysis on social functioning

As Fig. 1 shows, when social skills are concerned, the aquatic interventions are favored with larger mean within-group changes than those in the control group although these were not found to be significantly different.

**Fig. 2:**
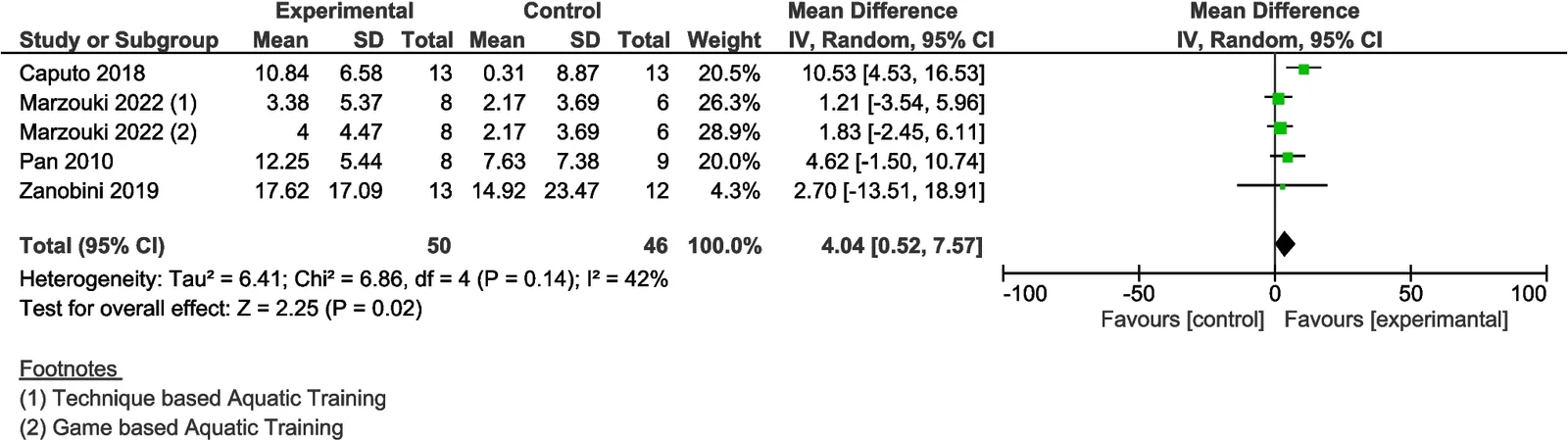
Meta-analysis on autistic behavior

For the meta-analyses on decreasing autistic behavior, the data from the total Childhood Autism Rating Scale (CARS) (Caputo et al., 2018), the stereotype subscale of the Gilliam Autism Rating Scale-2 (GARS-2) (Marzouki et al., 2022), the anti-social behavior scale of the SSBS-2 (Pan, 2010), and the total scores of the Autism Behavior Checklist (ABC) (Zanobini & Solari, 2019) were used. As shown in Fig. 2, the results of the experimental group favor the control group significantly.

### Study Methodology and Level of Evidence

Table 2 shows the findings of the best evidence synthesis. The final column shows studies that do not put weight on the syntheses but are supportive of the outcome showing positive within-group changes.

## Discussion

This systematic review aimed to investigate the effects of aquatic interventions in children with ASD using best evidence synthesis and meta-analyses. This resulted in moderate evidence for positive effects on water- and land-related motor skills and on decreased autistic behavior. The meta-analysis showed a significant difference in autistic behavior in favor of the intervention group compared to the control group. Limited evidence was found on social functioning and meta-analysis showed larger means within the intervention group but no significance.

A strength of this review is that the included studies were published in a wide range of journals varying from aquatic-oriented, to therapy-based, and to specialized in autism. This resulted in a comprehensive selection of interventions that were developed from different perspectives and backgrounds. Characteristics of the included interventions were compared to identify similarities and differences to provide insight into the conceptualizations, terminology, and outcomes, gaining more insight into the active components of the intervention.

Before synthesizing the outcomes, it was necessary to determine to what extent the interventions could be compared. Despite the heterogeneity implied by the names and aims of the interventions, their content and structure were largely similar. For example, within all interventions, activities related to water adjustment, such as those performed in a vertical position, were alternated with preparatory swimming activities in a horizontal position (aquatic skills), like floating and kicking. Most interventions offered a structure of warming up and cooling down, with activities tailored to the child’s needs in between. This is in line with the findings that indicate that routine and structure provide improved learning opportunities for children with ASD (McDougal et al., 2020). All studies used standardized assessments for assessing motor and social skills, except for one study (Alaniz et al., 2017) using the Goal Attainment Scaling (GAS), measuring personal learning goals of the children. Measuring personal goals for children may be more sensitive to change compared to standardized assessments (McDougall & Wright, 2009; Ruggeri et al., 2020). Nearly all interventions offered opportunities for playing or game-based activities. Learning through play is a didactic approach that may contribute to motor and social skill development in children with ASD (Gibson et al., 2021; Hassani et al., 2022; O’Keeffe & McNally, 2023). Another similarity is the use of sensory learning support, of which the visual and physical ones were mentioned most often. Lastly, all interventions have in common that at least one professional involved in the intervention was either a trained therapist or teacher with years of experience in special educational needs. Additionally, each intervention involved at least one professional with extensive experience in providing aquatic therapy or swim training to children. This suggest that all studies ensured that both behavioral and aquatic functioning were professionally influenced in their interventions.

It is noticeable that all studies included in the best evidence syntheses and meta-analyses, except Fragala-Pinkham et al. (2011), indicate that their method is based on the Halliwick concept. This concept aims at teaching mental adjustment, rotation control, and movement to learn to function as independently as possible in the water. Assuming that independence is a prerequisite for participation in divers aquatic contexts (Lambeck, 2015; Martin, 1981). Such similarity was not found in the studies with controls on social skills and autistic behavior. Overall, we can conclude that the aquatic interventions of the included studies are fairly homogeneous and therefore their outcomes can be synthesized.

All 17 studies on motor outcomes reported positive changes in motor skills. Given the fact that children with ASD often experience difficulties in adapting to new situations (Operto et al., 2021; Pugliese et al., 2016), these results suggest that the necessary motor skills for safety and enjoyment in the water can be effectively taught to children with ASD within reasonable timeframe. Six of these studies had a control group and were therefore candidates for best evidence syntheses. If best evidence syntheses had been conducted on all of these six CCTs together, strong evidence would have been found. However, due to the heterogeneity of the measured motor skills, for best evidence syntheses, we had to specify the outcomes on motor skills into land- and water-related, and the latter in two categories related to horizontal (adjustment skills) or vertical movements (aquatic skills), because they require different skills. Four of the CCTs reported significant differences in favor of the aquatic intervention: Ansari et al. (2021) on balance, Marzouki et al. (2022) on locomotor and object control skills, and Pan (2010) and Pan (2011) on water adjustment and aquatic skills. The other two controlled studies (Chu & Pan, 2012; Fragala-Pinkham et al., 2011) did not find any significant differences between the groups. Factors that may have influenced the non-significant findings regarding the outcome on aquatic skills are, for example, the method of measurement. In the study of Fragala-Pinkham et al. (2011), the water-related motor skills were assessed by children’s parents via a questionnaire, whereas in the other studies, the aquatic skills were measured by trained professionals in the aquatic context. It is unclear if the parents’ observation skills were sufficient to get reliable information. Regarding the study of Chu and Pan (2012), all three groups were offered the same aquatic activities but with different guidance styles. This may have resulted in a lack of contrast between the groups which could potentially explain the non-significant results.

It seems reasonable that aquatic adjustment or aquatic skills can be learned only in a context with water-specific characteristics. This raises the question of whether comparing aquatic skills in groups with and without an aquatic invention is a valid comparison. Therefore, the results of these studies using water-specific outcomes should be interpreted with caution as generalizability to land-related motor skills could be limited. However, these results do suggest that safe participation in aquatic sports, leisure, and family activities in children with ASD is feasible and might lead to increased physical activity and social interaction possibilities (Holloway et al., 2018; Kruger et al., 2019; Oliveira et al., 2021).

Regarding the land-related motor skills, the positive significant between-group effects that were found in two studies (Ansari et al., 2021; Marzouki et al., 2022) are important to mention. These studies support the hypothesis that aquatic interventions contribute to an improvement of motor skills in general. Both studies found significant differences on respectively balance and gross motor functioning compared to controls undergoing therapy as usual or engaging in regular sports activities. Both the aquatic intervention group and the Kata group in the study of Ansari et al. (2021) improved significantly on balance in 10 weeks, compared to the controls with TAU. This is notable because the activities in the aquatic intervention group were not specifically aimed at balance whereas Kata interventions exclusively target balance (Gauchard et al., 2018; Margnes & Paillard, 2011). This suggests that aquatic interventions play a role in implicitly improving balance. This implicit improvement of land-related motor skills by aquatic interventions is also seen in the study of Marzouki et al. (2022). Their 8-week aquatic interventions showed significantly improved locomotor and object control skills in both the Halliwick and the swim technique group, compared to the controls doing sports as usual. This is consistent with studies that have reported improvements in balance and motor skills after an aquatic intervention in children and adults with neurological disorders (Carroll et al., 2020; Chandolias et al., 2022; Fatorehchy, 2019; Montagna et al., 2014; Terrens et al., 2020; Veldema & Jansen, 2021). These findings are valuable as they suggest a positive transfer of learned water-related motor skills to land-related balance and locomotion which, in turn, can contribute to reducing the risk of motor impairments in children with ASD (Mickle et al., 2011; Stins & Emck, 2018). For future studies, we recommend conducting high-quality, controlled research that compares water- and land-related motor skills and their correlation.

The effects of aquatic interventions on social skills are less consistent when compared to those on motor skills. There are various factors contributing to this inconsistency. First factor is that social skills are a complex concept involving different aspects of cognitions, emotions, and behavior (Storebø et al., 2010). The included 11 studies with outcomes on social skills reported their outcomes in various aspects of social skills such as social interaction, social competence, autistic behavior, and/or quality of life. Second, these studies used 13 different measurement instruments which made comparability cumbersome. Therefore, we divided the outcomes for best evidence syntheses and meta-analysis in social skills and autistic behavior. This has resulted in limited effect on social skills and moderate effect on autistic behavior.

Five of the 11 studies on social and autistic behavior (Caputo et al., 2018; Chu & Pan, 2012; Marzouki et al., 2022; Pan, 2010; Zanobini & Solari, 2019) were eligible for best evidence syntheses and meta-analysis. All these studies reported significant positive effects on social skills and decreased autistic behavior, favoring the experimental group. Although significant differences were found in a variety of subscales, none of the included studies reported a significant difference in the total scale except for the study of Chu and Pan (2012). The latter specifically measured social interaction during the intervention. The other studies used instruments measuring more aspects of social skills in a broader context.

For the meta-analyses on social skills, the same studies were used except for Chu and Pan (2012) as their controls also received an aquatic intervention. The meta-analysis on social skills favored the experimental interventions although the difference between the intervention and control group did not reach significance. This result can be explained by three factors. First, the study that showed an overall significant improvement in social interaction (Chu & Pan, 2012) was not included in the meta-analysis as the control groups did not fit our criteria, since they also received aquatic interventions. Second, the interventions in the included studies, except for the intervention in Chu and Pan (2012), were aimed at practicing water adjustment, aquatic skills and play, and not explicitly at practicing social skills. While to improve social skills in children with ASD, a social skill-related, didactic teaching approach seems to be most effective (Wolstencroft et al., 2018). In addition, the intervention of Chu and Pan (2012) was the only one using explicit interaction with neurotypical children. Zanobini and Solari (2019) offered the opportunity to play with peers but the other studies did not use peer-related play to support learning the basic rules and actions of social interaction through observations and imitations of neurotypical peers as suggested by other studies (Adolph & Hoch, 2019; Derikx et al., 2021; Lee et al., 2022; Livesey et al., 2011). Third, the control group in the study of Zanobini and Solari (2019), included in the meta-analyses, received alternative sports and psycho-educational interventions. If these controls had had therapy as usual, the comparison would have been more objective since both conditions in that study showed improvement in social skills, although the intervention group scored significantly better on social motivation.

Best evidence synthesis and meta-analysis on autistic behavior were performed to gain more insight into the change of social skills because autistic behavior is characterized by impairments in social interaction and communication and by repetitive stereotypical behavior (American Psychiatric Association, 2013). The latter is considered a relevant barrier for social communication (Lee et al., 2007) and learning ability (Cook & Rapp, 2020; Haghighi et al., 2022). Four studies (Caputo et al., 2018; Marzouki et al., 2022; Pan, 2010; Zanobini & Solari, 2019) were included in the best evidence syntheses on autistic behavior which showed moderate evidence for the effects of aquatic interventions. Pan (2010) and Marzouki et al. (2022) reported significant differences on the total scale, the other two studies only on subscales. The meta-analysis on autistic behavior, based on the same four studies, showed a significant difference in favor of the intervention group suggesting that aquatic activities decrease autistic behavior. This could be important since decreased autistic behavior is associated with increased participation in activities (Haghighi et al., 2022). This finding is also in the interest of motor development because a strong correlation was found between motor impairment and autistic characteristics (Bhat, 2021).

The difference between the outcomes of best evidence synthesis and meta-analysis on autistic behavior can be explained by the use of more data in the meta-analyses compared to the data that were used in the single studies. The two studies (Caputo et al., 2018; Zanobini & Solari, 2019) that showed no significant differences between the conditions both reported significant positive changes within the intervention group with smaller change in the accompanying control conditions. Another explanation could be that in the meta-analysis, subscales specifically related to autistic behavior were compared instead of the total scales, whereas in the best evidence syntheses, the results of the total summarized scales were compared.

Altogether there is a positive trend that an aquatic intervention improves social skills and decreases autistic behavior. These effects appear to be generalizable to different contexts because the questionnaires were mainly completed by parents, caregivers, and teachers (Alaniz et al., 2017; Caputo et al., 2018; Güeita-Rodríguez et al., 2021; Marzouki et al., 2022; Mills et al., 2020; Oriel et al., 2020; Zanobini & Solari, 2019). It remains unclear which aspects of social skills is most affected by aquatic interventions, due to heterogeneous methodologies and measurement instruments. For future research, we recommend researching specific aspects of social skills affected by aquatic interventions and additional if and how these aspects relate to the assessed motor skills.

### Limitations

Regarding this review, there are few limitations that should be addressed. Despite efforts made to gain extra information on unpublished research, e.g., via clinicaltrials.org, no unpublished studies could be included. Therefore, publication bias could not be avoided, as only studies accepted for publication could be used.

The sample sizes of the included controlled studies were relatively small. Although very common in clinical studies in ASD (Lombardo et al., 2019), this can be a significant source of bias and can make it difficult to extrapolate the results to the overall population of children with ASD (Faber & Fonseca, 2014). Further, the male-to-female ratio in the included studies was 9:1. This differs from the assumed 3 to 4:1 ratio in ASD (Loomes et al., 2017). Therefore, girls with ASD are underrepresented in this review which limits the generalizability of the outcomes to girls in this systematic review. For future research, we recommend a better gender distribution.

To achieve a balanced perspective in a review, adverse aspects of the intervention and dropouts should be taken into account (Higgins et al., 2019). It is expected that most children with autism have difficulty adapting to new situations (Bertollo et al., 2020; Cook & Rapp, 2020; Pugliese et al., 2016), which may increase the risk of adverse events. None of the included studies in our review reported the presence of adverse events related to motor or social skills. We recommend systematically monitoring adverse events and dropouts in future research to get more insight in possible adverse events and how to prevent them.

## Conclusion

### Implications for Practice

Evidence suggests that structured aquatic interventions guided by both professionals in influencing behavior and swimming skills and adapted learning support can positively improve water- and land-related motor skills in children with ASD. Moderate overall evidence on this type of change was found. This review showed that children with ASD can learn motor skills that help them to feel comfortable and adjusted in a pool context, and positively affect their balance and locomotion on land. Limited evidence was found on the effect of aquatic interventions on social skills, but remarkable is the significant decrease of autistic behavior in the intervention groups compared to the control groups. Overall, the results suggest that an aquatic intervention might lead to improved social skills in children with ASD.

### Implications for Future Research

To show effect from aquatic interventions on motor or social skills in children with ASD, more comparable and controlled quality research is needed. Homogeneity of design and measurement instruments can contribute to this. Studies should use calculated adequate sample sizes, with representative proportions of boys and girls, and include both intervention and active control conditions. To get a better understanding of the effects of an aquatic intervention on motor skills, both water- and land-related motor skills should be measured, and compared. To get a better understanding of the association between improved motor skills through aquatic interventions and social skills, further research is necessary.

## Figures and Tables

**Fig. 3:**
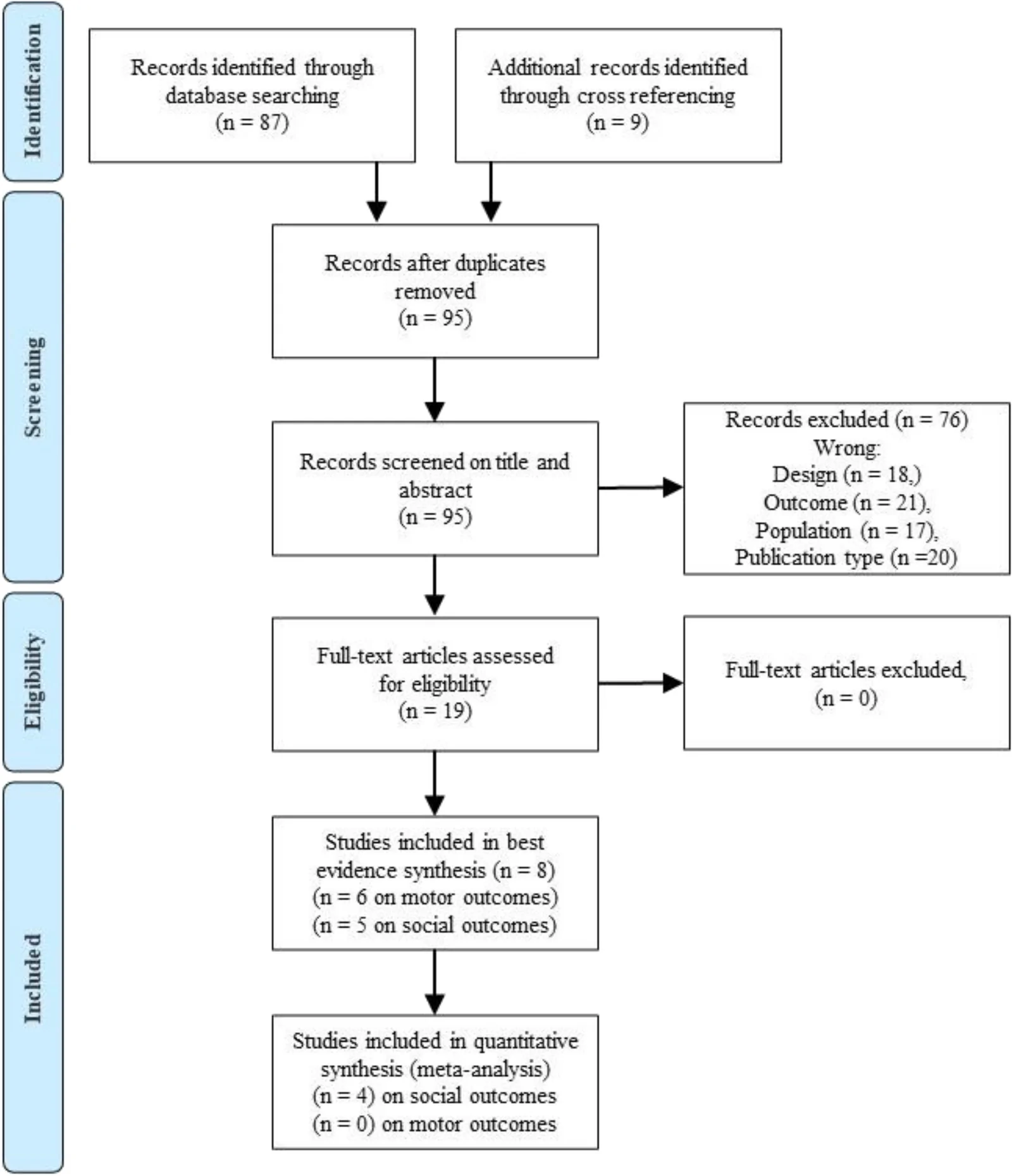
PRISMA Flow chart

**Table 3:**
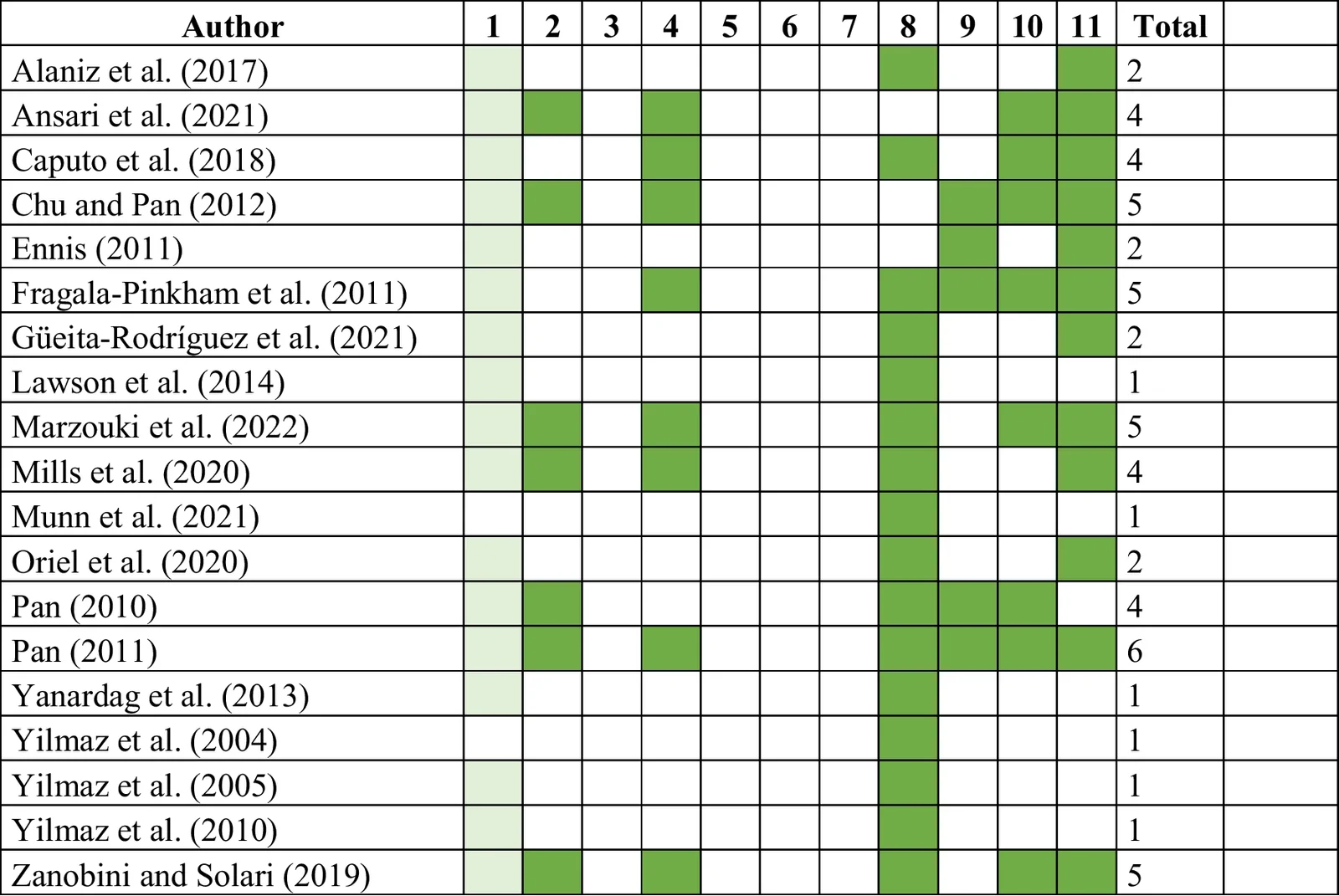
Measurement of PEDro methodical quality

1. Eligibility criteria 2. Random allocation 3. Concealed allocation 4. Baseline comparability 5. Blind subjects 6. Blind therapists 7. Blind assessors 8. Measure of one key outcome 9. Intention-to-treat analysis 10. Between-group comparisons 11. Point estimates and variability. Eligibility criteria item 1 does not contribute to the total score

## Appendix 1

Please see Fig. 3.

## Appendix 2

Please see Table 3.

## Appendix 3

Please see Table 4.

**Table 4:** Funding of the included articles

Author	Funding
Alaniz et al. (2017)	Casa Colina Centers for Rehabilitation Foundation.
Ansari et al. (2021)	Not mentioned
Caputo et al. (2018)	Not mentioned
Chu and Pan (2012)	National Science and Technology Council (Taiwan)
Ennis (2011)	Not mentioned
Fragala-Pinkham et al. (2011)	Thoracic Foundation, John W. Alden Trust and Innovating Worthy Projects Foundation
Guéita-Rodriguez et al. (2021)	No external funding
Lawson et al. (2014)	Autism speaks Family Community Grants
Marzouki et al.(2022)	No specific grant from any funding agency
Mills et al.(2020)	No external funding
Munn et al.(2021)	No external funding
Oriel et al. (2020)	Not mentioned
Pan (2010)	National Science and Technology Council (Taiwan)
Pan (2011)	National Science and Technology Council (Taiwan)
Yanardag et al. (2013)	Grant from Anadolu University Fund
Yilmaz et al. (2004)	Not mentioned
Yilmaz et al. (2005)	Not mentioned
Yilmaz et al. (2010)	Not mentioned
Zanobini and Solari (2019)	Not mentioned
